# Paradoxical psoriasis after the use of anti-TNF in a patient with rheumatoid arthritis*

**DOI:** 10.1590/abd1806-4841.20164456

**Published:** 2016

**Authors:** Jaqueline Barbeito de Vasconcellos, Daniele do Nascimento Pereira, Thiago Jeunon de Sousa Vargas, Roger Abramino Levy, Geraldo da Rocha Castelar Pinheiro, Ígor Brum Cursi

**Affiliations:** 1 Universidade do Estado do Rio de Janeiro (Uerj) – Rio de Janeiro (RJ), Brazil; 2 Hospital Geral de Bonsucesso (HGB) – Rio de Janeiro (RJ), Brazil

**Keywords:** Interferons, Psoriasis, Therapeutics, Tumor necrosis factor-alpha

## Abstract

The use of tumor necrosis factor antagonists (anti-TNF) has become a usual practice to treat various inflammatory diseases. Although indicated for the treatment of psoriasis, anti-TNF may paradoxically trigger a psoriasiform condition. We present a case of a female patient who, during the use of infliximab for rheumatoid arthritis, developed psoriasis. In an attempt to switch anti-TNF class, we observed a cumulative worsening of the lesions requiring suspension of the immunobiological agent and the introduction of other drugs for clinical control. The therapeutic challenge of this paradoxical form of psoriasis is the focus of our discussion. The use of another anti-TNF in these patients is a matter of debate among experts.

## INTRODUCTION

Psoriasis is a chronic immune-mediated inflammatory skin disease with a genetic component and a wide range of clinical manifestations. It affects around 1%-2% of the world’s population.^1^ The use of biopharmaceuticals – especially tumor necrosis factor antagonists (anti-TNFs) – has become common practice in the treatment of various inflammatory diseases such as psoriasis. Side effects of this therapy include new forms or change in the pattern of the psoriasis lesions or even worsening of symptoms, in patients with or without prior psoriasis or psoriatic arthritis. This phenomenon is called paradoxical psoriasis. It occurs in approximately 5% of patients using anti-TNF drugs. Although the disorder affects both sexes, it has a slight predilection for women.^1-4^ The time between the introduction of the medication and the appearance of lesions can range from a few days to many months. The most commonly reported clinical forms are palmoplantar pustular psoriasis followed by plaque-type psoriasis and guttate psoriasis.^5^ Nail and scalp involvement have also been described. Some patients may experience more than one type of lesion. The treatment of the paradoxical phenomenon of psoriasis is still a challenge because most patients need anti-TNF inhibitors to control the underlying disease. We report a patient using anti-TNF inhibitors who developed paradoxical deterioration after switching to another medication of the same class.

## CASE REPORT:

**Figure 1:**
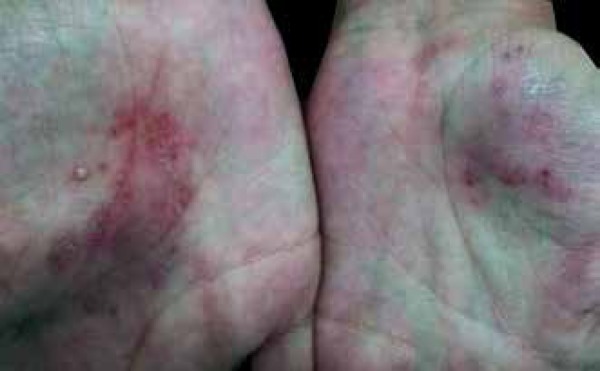
Initial clinical picture. Detail of bilateral palmar region

**Figure 2:**
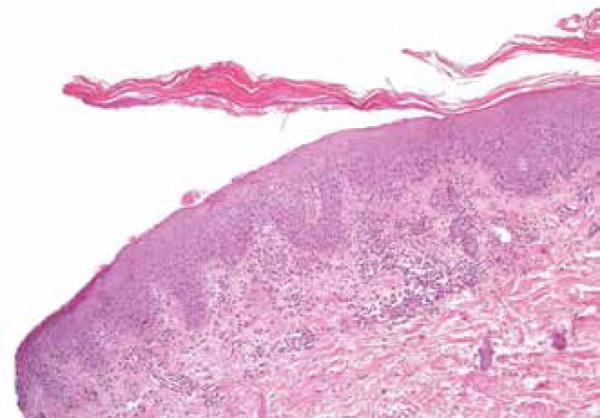
Histopathology. Skin with mild to moderate psoriasiform hyperplasia, absence of granulose layer, and parakeratosis permeated by neutrophils. This histopathological pattern is found in guttate or eruptive psoriasis lesions (HE, 100x)

**Figure 3:**
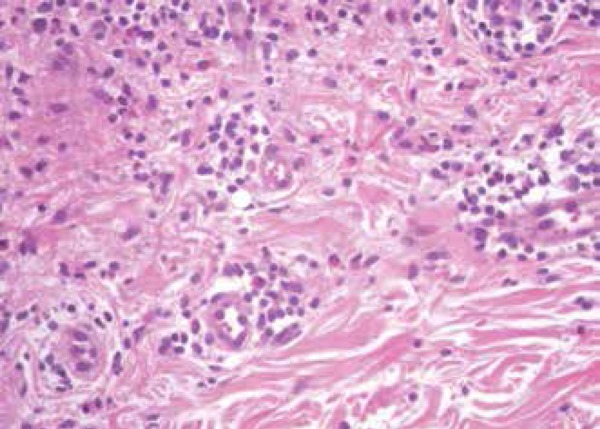
Histopathology. Perivascular inflammatory infiltrate consisting of lymphocytes, neutrophils, and eosinophils. The presence of eosinophils in the infiltrate of a psoriasis lesion can occur when it is triggered by drugs. (HE, 400x)

**Figure 4:**
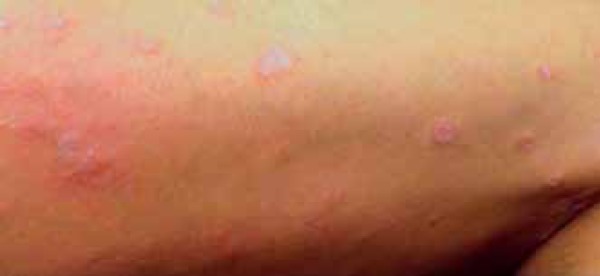
Clinical picture after switching to adalimumab. Detail of the right lower limb

**Figure 5:**
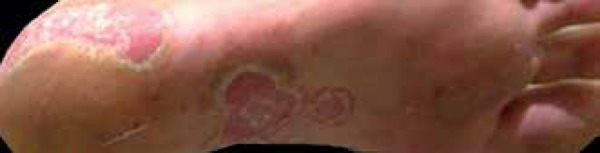
Clinical picture after switching to adalimumab. Detail of the right plantar region

**Figure 6:**
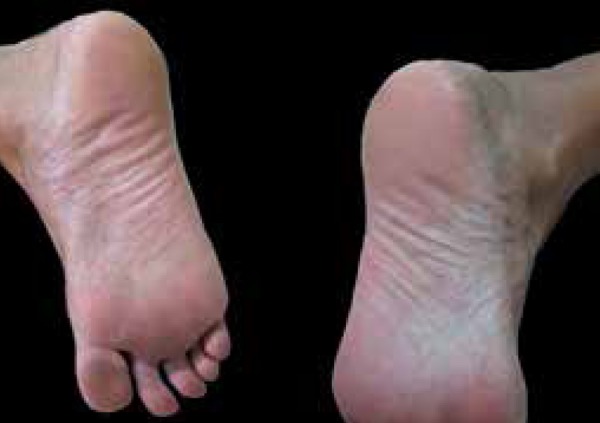
Clinical picture after anti-TNF suspension. Detail of the bilateral plantar region

We report a 43-year-old white female patient, married, born and raised in Rio de Janeiro, diagnosed with rheumatoid arthritis since 2006. She was treated with prednisone (5 mg/daily) and leflunomide (20mg/daily) without satisfactory response. She started using infliximab (300 mg – 5 mg/kg – every eight weeks) and, 10 months later, presented with erythematous plaques distributed on the trunk, back, and limbs. She also reported erythematous, scaly plaques with some pustules on the feet and hands and scaling on the scalp (Figure 1). Histopathological examination results of one of the lesions were consistent with psoriasis: moderate psoriasiform epidermal hyperplasia, absent stratum granulosum, and parakeratotic stratum corneum permeated by neutrophils associated with perivascular and superficial inflammatory infiltrate consisting of lymphocytes, neutrophils and eosinophils (Figures 2 and 3). We switched from infliximab to adalimumab. Two weeks later, we observed a worsening of the lesions with increased palmoplantar scaling, erythematous scaly plaques, and pustules on the lower limbs (Figures 4 and 5). We suspended adalimumab and started with cyclosporine (200 mg/daily – 3 mg/kg/day), with subsequent clinical improvement. However, we still observed mild palmar scaling and nail pitting. Because of the uncontrolled hypertensive peak that required hospitalization, we suspended cyclosporine. Four weeks after the start of methotrexate (7.5 mg/week), there was a worsening of the skin lesions. Noting no improvement, even after increasing the dose, we also suspended methotrexate. Six weeks after the reintroduction of leflunomide, we observed a remission of the skin lesions (Figure 6).

## DISCUSSION:

Paradoxical psoriasis occurs more frequently in patients who are using infliximab and suffer from rheumatoid arthritis.^5^ The etiology of this manifestation is not well-defined, but seems to be related to the balance between the levels of tumor necrosis factor alpha (TNF-alpha) and interferon (IFN), with increased levels of the latter cytokine.^5,6^ Gannes *et al.* suggested that inhibition of TNF-alpha may stimulate uncontrolled production of interferon-alpha (IFN-alpha), which in certain patients can induce the development of the disease. The authors also showed that the expression and activity of IFN-alpha were increased in patients who develop psoriasis during treatment with anti-TNF drugs compared to patients with previous psoriasis vulgaris.^7^ The treatment of psoriasis induced by anti-TNF drugs depends on the severity of the clinical picture and still represents a therapeutic challenge. In milder cases, topical treatment and phototherapy can be prescribed in an attempt to control the skin condition em vigência do immunobiological agent, which is often the only effective drug for the treatment of the underlying disease. However, in most cases (about 75% of cases) a systemic treatment is required, with cyclosporine, acitretin, and methotrexate use reported. The suspension of the implicated biologic agent is associated with a higher resolution rate, but often the suspension alone is not enough. The suspension of the anti-TNF drug associated with the start of systemic medication for psoriasis resulted in higher cure rates: approximately 64% versus 44% when systemic medications started without suspension of the immunobiological agent.^5,8,9^ Thus another type of anti-TNF agent can be used for the treatment of the underlying disease. One study showed an improvement of the skin condition in 15% of cases when anti-TNF medications were switched.^5^ However, paradoxical psoriasis may be persistent or even get worse, as occurred with our patient. In most cases, no improvement in skin condition is reported, suggesting a class effect, and not the effects of a specific medication.^5^ In this context, treatment of the underlying disease becomes a challenge, since the anti-TNF drug is often the only effective treatment for the underlying disease. In the present case report, the presence of eosinophils in histopathology suggested medication as a trigger, which explains our option to switch anti-TNF agents.^10^ Our patient, however, showed worsening of the lesions, suggesting a class effect and the need for an immunobiological agent class switching. Ustekinumab, anti-IL-12 antibody, and anti-IL-23, have played a prominent role in psoriasis treatment by blocking an important path in the pathogenesis of the disease: the production of Th1 and Th17 CD4 cells. Its use in the treatment of rheumatoid arthritis and psoriatic arthritis is still off-label, but it is an alternative to the control of the disease when other medications can not be used.^3^ In the reported case, the patient was able to achieve adequate control of rheumatoid arthritis with non-immunobiological drugs, a fact that was decisive for the regression of the psoriatic lesions.
